# Interplay of Various Evolutionary Modes in Genome Diversification and Adaptive Evolution of the Family *Sulfolobaceae*

**DOI:** 10.3389/fmicb.2021.639995

**Published:** 2021-06-25

**Authors:** Rachana Banerjee, Narendrakumar M. Chaudhari, Abhishake Lahiri, Anupam Gautam, Debaleena Bhowmik, Chitra Dutta, Sujay Chattopadhyay, Daniel H. Huson, Sandip Paul

**Affiliations:** ^1^Structural Biology and Bioinformatics Division, CSIR-Indian Institute of Chemical Biology, Kolkata, India; ^2^Academy of Scientific and Innovative Research (AcSIR), Ghaziabad- 201002, India; ^3^Department of Pharmacoinformatics, National Institute of Pharmaceutical Education and Research, Kolkata, India; ^4^JIS Institute of Advanced Studies and Research, JIS University, Kolkata, India; ^5^Institute for Bioinformatics and Medical Informatics, University of Tübingen, Tübingen, Germany; ^6^Cluster of Excellence: Controlling Microbes to Fight Infection, Tübingen, Germany

**Keywords:** thermoacidophilic archaea, pan-genome, genome evolution, positive selection, metabolic pathways

## Abstract

*Sulfolobaceae* family, comprising diverse thermoacidophilic and aerobic sulfur-metabolizing Archaea from various geographical locations, offers an ideal opportunity to infer the evolutionary dynamics across the members of this family. Comparative pan-genomics coupled with evolutionary analyses has revealed asymmetric genome evolution within the *Sulfolobaceae* family. The trend of genome streamlining followed by periods of differential gene gains resulted in an overall genome expansion in some species of this family, whereas there was reduction in others. Among the core genes, both *Sulfolobus islandicus* and *Saccharolobus solfataricus* showed a considerable fraction of positively selected genes and also higher frequencies of gene acquisition. In contrast, *Sulfolobus acidocaldarius* genomes experienced substantial amount of gene loss and strong purifying selection as manifested by relatively lower genome size and higher genome conservation. Central carbohydrate metabolism and sulfur metabolism coevolved with the genome diversification pattern of this archaeal family. The autotrophic CO_2_ fixation with three significant positively selected enzymes from *S. islandicus* and *S. solfataricus* was found to be more imperative than heterotrophic CO_2_ fixation for *Sulfolobaceae*. Overall, our analysis provides an insight into the interplay of various genomic adaptation strategies including gene gain–loss, mutation, and selection influencing genome diversification of *Sulfolobaceae* at various taxonomic levels and geographical locations.

## Introduction

The microbial genome divergence is a dynamic evolutionary process of ecological and genetic differentiation. Various mechanisms that both create and maintain the phenotypic diversity in genomes include gene gain–loss, mutation, natural selection, and genetic drift (Loewe and Hill, 2010). Among these, the gene gain–loss is considered to be one of the important factors affecting the microbial evolution, and further gene gain–loss along with habitat isolation are proposed to be the key points of ecological speciation in microbes (Polz et al., 2013). Several other studies have also shown that ecological divergence and natural selection may play important roles in genome diversification (Vos, 2011; Shapiro et al., 2016). Geographical isolation along with genetic drift also impacts the biogeographic patterns of microorganisms and is responsible for microbial biodiversity (Martiny et al., 2006; Andam et al., 2016).

A considerable fraction of the genetic diversity is likely to be considered as regulators of adaptive features specific for that particular habitat. Deciphering these adaptive signals encrypted in the genomes of any organism is important from both evolutionary and ecological perspectives. The complex nature of these signals due to the multilayer interactions either with cohabitants or with environment makes the task extremely challenging. In this context, one important module of research is the evolutionary dynamics of extremophiles that offer insights into how their genome-wide variations, linked to structural and functional profiles, are shaped by extreme environmental attributes (Horikoshi and Grant, 1998; Seckbach, 2000). Extremophiles can withstand and flourish in environments that are otherwise considered to be too extreme to support any terrestrial life forms. Extreme environments are generally characterized by extreme temperatures, pressures, pH, and salinity (Rothschild and Mancinelli, 2001). The archaeal domain, being a substantial part of the extremophiles, has specifically evolved and acquired adaptations to sustain such extreme environments (Reed et al., 2013). Availability of high-quality, large-volume, and low-cost genomic data has augmented the study to deal with the global properties of the genomes and helped to reveal the various adaptive mechanisms to survive in extreme environments. During the past decade, several studies have reported on the influence of genomic attributes including relative synonymous codon usage bias, dinucleotide abundance, purine loading, and horizontal gene transfers (HGTs) on sustaining extreme conditions (Lynn et al., 2002; Das et al., 2006; Paul et al., 2008; Dutta and Paul, 2012; van Wolferen et al., 2013).

One powerful approach to unraveling the molecular mechanisms of adaptation is the comparative genomics study with a large number of complete genome sequences. In this regard, the pan-genome approach has been found to be valuable in the comparative analysis for a set of microbial genomes (Lefébure and Stanhope, 2007; Tettelin et al., 2008). Conventionally, the entire collection of genes present in diverse strains of a particular species is termed as “pan-genome” (Tettelin et al., 2008; Brockhurst et al., 2019). On the one hand, site-specific recombination circulated through mobile genetic elements and gene gain–loss events are associated with the flexible or accessory genes and thereby offer genomic and metabolic diversity in a population; the core genomic part, on the other hand, is linked with vertical transmission and homologous recombination, providing a stable genomic and metabolic backbone for the population. As the core clusters embody a set of highly conserved genes, phylogenetic analysis on the basis of core gene clusters provides information on the evolutionary history of its constituent organisms (Lefébure and Stanhope, 2007; Chattopadhyay et al., 2012a; Freschi et al., 2019). Thus, comparative pan-genomic study coupled with evolutionary analyses could be used as a framework to investigate the genome diversification associated with life under extreme conditions.

In the present study, we have used a comprehensive set of extremophilic Archaea from the family *Sulfolobaceae*. The family consists of thermoacidophilic organisms, with optimal growth temperatures ranging from 65 to 90°C and optimum growth at pH values of approximately 2. Another notable feature is the aerobic sulfur oxidation, which is restricted to the members of this family among Archaea (Friedrich et al., 2005; Wu et al., 2021). Several studies illustrate *Sulfolobus*, *Saccharolobus* (*Saccharolobus solfataricus* previously *Sulfolobus solfataricus*) *Acidianus*, and *Metallosphaera* as the principal components and enriched consortium of the microbial communities from hot springs (Kozubal et al., 2008; Sakai and Kurosawa, 2018). The genus *Sulfolobus* has enormous importance from biotechnological viewpoint as members of this genus are a source of new enzymes, biomaterials, and metabolic pathways and also efficient in oxidative desulfurization of sour crude oil and have a role in decreasing the cost of processing of petroleum (Benvegnu et al., 2009; Bräsen et al., 2014; Besse et al., 2015; Littlechild, 2015; Nazari et al., 2017; Quehenberger et al., 2017). Also, the most commonly studied sulfur-oxidizing genera *Sulfolobus/Saccharolobus* comprised species displaying a wide variety of genomic characteristics. Despite being biologically quite similar and dwelling in comparable environments, there are obvious differences between *Sulfolobus islandicus* and *Sulfolobus acidocaldarius* strains (Mao and Grogan, 2017). For instance, among *S. islandicus* isolates, through a comparative genomic study, it has been observed that geographical isolation plays a significant role in its increased genomic diversity between populations (Reno et al., 2009). Several studies indicate that genomes diverge genetically and metabolically according to the geographic locations, supporting the fact that both core and accessory parts are restricted because of discontinuous habitat (Whitaker, 2003; Mao and Grogan, 2017). On the other hand, for another species of this genus *S. acidocaldarius*, the genome is remarkably conserved across different geographical locations, and the evolutionary basis for this inconsistency in genome diversity of *S. acidocaldarius* with *S. islandicus* is yet to be understood. Previous works have discussed several factors that account for the high preservation among geographically different *S. acidocaldarius* strains, for example, propagule dispersal, differential ability to colonize and replace local populations, resistant nature toward mobile elements, etc. (Brügger et al., 2002; Chen et al., 2005; Redder and Garrett, 2006; Anderson et al., 2017; Mao and Grogan, 2017; Wagner et al., 2017).

Thus, the complete genomes of the isolates from the family *Sulfolobaceae* offer an excellent opportunity to grasp the role of HGT, recombination, and mutation in their differential genomic and metabolic diversification in presence or absence of selection pressures. Toward this, we performed a comparative pan-genomic and subsequent evolutionary analysis for publicly available complete genomes of the members of family *Sulfolobaceae* and deciphered the contingent contributions of gene gain–loss, mutation, and recombination in shaping up the asymmetric evolution of this archaeal family. We believe that our study sheds light on the understanding of fundamental evolutionary processes along with the selective pressures that have shaped genomic and metabolic diversification of the family *Sulfolobaceae*.

## Materials and Methods

### Sequence Retrieval and Annotation

Fully assembled chromosomes of 30 organisms belonging to the family *Sulfolobaceae* including genera *Sulfolobus* (15 strains), *Saccharolobus* (6 strains), *Metallosphaera* (7 strains), and *Acidianus* (2 strains) from different geographical locations were considered from the NCBI GenBank resource^1^ (Supplementary Table 1). We extracted the genome annotations available in NCBI for all the 30 genomes used in the present study. We also reannotated the genomes using the RAST annotation server (Aziz et al., 2008) and compared them with the annotation extracted from NCBI. Both the annotations were found to be 99.01% ± 0.68% identical. We used NCBI-provided annotations for further analysis.

### Pan-Genome Construction

For pan-genomic profile construction, we used all the annotated protein coding genes (total 76,369) from 30 genomes under study (Supplementary Table 1). We implemented CD-HIT Suite (Fu et al., 2012) for clustering of all the amino acid sequences by considering 50% sequence identity and alignment coverage as orthologous clustering criteria (Edgar, 2010; Li et al., 2012; Steinegger and Söding, 2018; Tonkin-Hill et al., 2020) to construct strain-wise gene presence/absence–abundance pan-matrix. We also used OrthoMCL (Li et al., 2003) for the identification of orthologous groups applying 50% sequence identity and alignment coverage and implemented GET_HOMOLOGUES software package (Contreras-Moreira and Vinuesa, 2013) for detailed pan-genome analysis along with the reconstruction of orthologous gene families. Both pan-matrices (derived from CD-HIT and OrthoMCL) were then used to identify and extract core/mosaic/unique clusters. In order to maintain uniqueness for core gene sets, in case of duplicates, only one sequence from each organism is considered in each gene cluster. In this way, we removed all paralogous and duplicate sequences. Then, we selected as core only those orthologous gene clusters detected as core gene families by both CD-HIT and OrthoMCL algorithms. In order to perform different evolutionary analyses, we extracted the nucleotide sequence of core clusters.

Apart from carrying out comparative genomics at the intergenus level considering four different genera of the same family *Sulfolobaceae*, we also carried out pan-genomic studies at species level separately for four species containing multiple strains, viz., *S. acidocaldarius*, *S. islandicus*, *S. solfataricus*, and *Metallosphaera sedula*. For reconstruction of pan-genomes for these four species separately, we followed the same steps as mentioned previously for gene clustering of the entire *Sulfolobaceae* family.

### Study of Recombination

We identified probable recombination events for each of the core clusters using the Recombination Detection Program (RDP4) default settings (Martin et al., 2015). This software includes an array of eight different recombination-detection algorithms. These algorithms are from three different methods: phylogenetic (BOOTSCAN, RDP, and SISCAN), substitution (GENECONV, MAXCHI, CHIMAERA, and LARD), and distance comparison (PHYLPRO). We assigned a gene region to be tentatively affected by homologous recombination event only by finding significant (*P* < 0.05) results in at least three out of these different recombination detection algorithms in RDP4.

### Phylogenetic Study

We used ClustalW (Larkin et al., 2007) to align amino acid sequences of core genes and FASconCAT (Kück and Meusemann, 2010) to concatenate the alignments. Two core phylogenies were created based on the variable regions of the concatenated alignments of all core genes as well as non-recombinant core genes, without considering the gaps by using Jones–Taylor–Thornton substitution model (Jones et al., 1992) under maximum-likelihood method of MEGA 7 (Kumar et al., 2016). The pan-genome phylogeny was constructed by performing a parsimony analysis in MEGA 7 by converting the gene presence/absence–abundance pan-matrix into a binary matrix and finally to hypothetical sequence by replacing 0 and 1 with A and T, respectively. All the phylogenetic profiles created in our study were statistically supported through 1,000 bootstrap iterations.

### Gene Gain–Loss Identification

In order to identify gene gains and losses, we used the COUNT (Csûös, 2010) software, applying asymmetric Wagner parsimony with gain penalty 1.6 and loss penalty 1. We considered the pan-genome phylogeny as the evolutionary trajectory of the genomes and the gene presence/absence–abundance pan-matrix as input in COUNT for gene gain–loss calculation. To confirm the COUNT results using a different approach, we implemented Sankoff’s parsimony algorithm (Sankoff, 1975). For each gene, ancestral presence (1) and absence (0) states were computed using a gain penalty of 1.6 and loss penalty of 1.0, favoring late occurrences of changes during trace-back. Based on Sankoff’s algorithm, for each node, the number of genes gained (state change from 0 to 1) and lost (state change from 1 to 0), and the number of genes present were computed in a tree traversal. The script implementing Sankoff’s parsimony algorithm was written in Python 3.8.0 and available on request.

### Functional Annotation and Metabolic Pathway Prediction

Functional annotations of selected genes were performed using Archaeal Clusters of Orthologous Genes (arCOGs) database (Makarova et al., 2015) (2014 update). We used the best BlastP hits (Camacho et al., 2009) for each representative protein sequence against arCOGs database. In order to retain the best hits, sequence identity cutoff of 50% with at least 50% query length coverage and *e*-value cutoff of 1.0E-5 were used. Functional annotations of selected genes for Kyoto Encyclopedia of Genes and Genomes (KEGG) Orthology (KO) assignments and prediction of KEGG pathways were performed using KEGG Automatic Annotation Server using Bidirectional Best Hits method with default parameters (Moriya et al., 2007). A χ^2^ test with Yates’ correction and two-tailed *P* values has been used to check the significant differences between the gain and loss for each arCOG and KEGG categories for a particular species (Supplementary Table 2). The *P* value stands for probability and quantifies how likely it is that any observed difference between groups is due to chance.

### Comparative Analysis of Metabolic Pathways

Literature survey and the information from the *Sulfolobus* Systems Biology (SulfoSYS) Project^2^ (Zaparty et al., 2010) were used to categorize 71 genes for Central Carbohydrate Metabolism (CCM). BlastP (50% sequence identity and alignment coverage) search of these 71 CCM genes against *Sulfolobaceae* unfiltered pan-genome yielded 2,329 homologs from 105 gene clusters with 71 different KO terms (Supplementary Table 3).

We identified a total of 135 and 12 genes for “Sulfur metabolism” and “Nitrogen metabolism,” respectively, from KEGG Pathway Database (Kanehisa and Goto, 2000). Homology search (as followed for CCM pathway) yielded 32 gene clusters (459 genes, 17 KO terms) and 14 gene clusters (191 genes, 11 KO terms) for sulfur and nitrogen metabolism, respectively (Supplementary Tables 4, 5). The abundance matrices for the three pathways were then converted into three single linkage clustering along with heatmaps by ClustVis web tool on the basis of Ward method using Manhattan distance (Metsalu and Vilo, 2015).

### Evolutionary Rate Calculation

We calculated the evolutionary rate dN/dS (ω) by considering all possible pairs (225,765) of 30 *Sulfolobaceae* genomes for each of the 519 core protein sequence clusters, using the “yn00” program (Yang and Nielsen, 2000) from Phylogenetic Analysis by Maximum Likelihood package. Synonymous sites and ω values were considered to be saturated or unreliable, when, dS < 0.1, or dN/dS ≥ 99, for a pair of sequences. Therefore, we discarded those pairs of sequences before calculating the average of ω values. Next, the average dN, dS, and dN/dS were calculated for all possible combinations at species and strain levels from 519 core genes.

### Identification of Positive Selection by the Branch-Site Method and Log-Likelihood Ratio Test

A total of 2,412 gene pairs (having dN > dS) comprising 354 protein sequence clusters were chosen as positively selected gene pairs out of the 225,765 gene pairs from the core group and subjected to further analysis through branch-site method (BSM) and log-likelihood ratio test (LRT) (Nielsen and Yang, 1998) for testing the statistical significance of positive selection along lineages. The lnL values of the 354 clusters were calculated for both the selection model and null model. The LRT values for each of the clusters were then calculated as follows: ΔLRT = 2 × (lnL1 – lnL0), where lnL1 represents the lnL values resulting from the selection model, and lnL0 represents the same from the null model (Vishnu et al., 2015). A significant result with the branch-site model using χ^2^ test (*df* = 1, *P* < 0.05) pointed toward the fact that positive selection has affected a particular cluster during a specific evolutionary time.

## Results and Discussion

### Genome Diversification and Differential Patterns of Gene Gain–Loss Events

**FIGURE 1:**
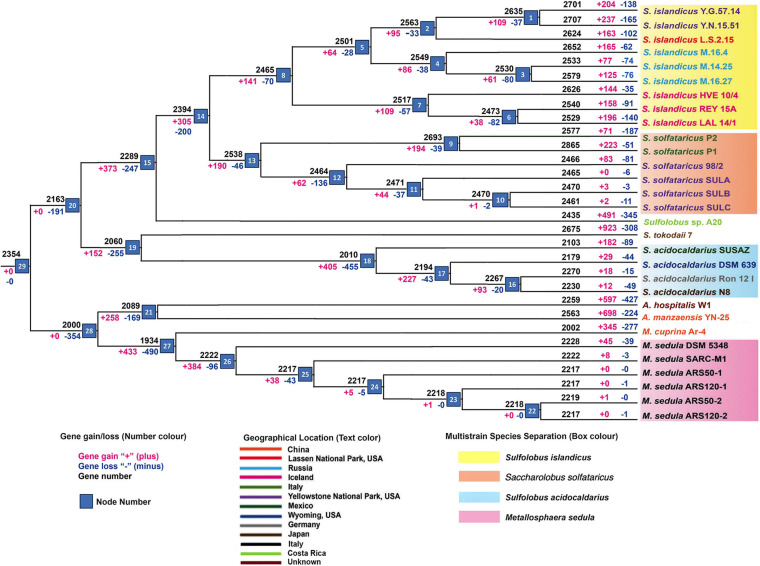
Summary of gene gain and gene loss events by applying asymmetric Wagner parsimony in the family *Sulfolobaceae*, across the phylogenetic tree.

The core genome phylogeny (Supplementary Figure 1A) constructed for the *Sulfolobaceae* family depicts the genome divergence pattern similar to that of the pan-genome tree (Supplementary Figure 1B) representing the balance of recent gene gains and losses in various ecosystems. Further weighted parsimony-based gene gain–loss estimation detected a set of 2,354 genes in the genome of the common ancestor for all members of the *Sulfolobaceae* family (Figure 1). There was an initial phase of genome shrinkage as the four genera began to diversify and the genome streamlining continued for most of the species groups. The internal or terminal nodes that were represented by either single strain or multiple strains of a particular species experienced an overall level of gene gain with one exception of *S. acidocaldarius*. At the strain level, this gene gain–loss dynamics varied a lot, where a majority of strains of a particular species experienced a large number of gene gains–losses at the terminal nodes, suggesting the acceleration of these events during the recent diversification of each strain. In general, terminal nodes were characterized by much higher frequency of gene gains than losses. In contrast, a significant amount of gene deletion was observed in case of two *S. acidocaldarius* strains (DSM 639 and N8) isolated from different geographic locations and *S. solfataricus* strain P2 from Italy. It should be noted that SULB and SULC resulted from extensive passage for an adaptive laboratory experiment from SULA; thus, they were practically the same strain with few genetic changes between them (McCarthy et al., 2015). The derivative strains for *S. solfataricus* and *M. sedula* showed primarily the gene loss events, implying restricted gene flow due to laboratory conditions and loss of function via mutation or deletion (Ai et al., 2016). Again, for all four genera, considerable amount of genome size variation of constituent species can be described by the differential phenomenon of gene acquisition and loss. Overall, the analysis outlined a dynamic gene gain–loss model through the genome diversification of *Sulfolobaceae* family. While *S. islandicus*, *S. solfataricus*, *Sulfolobus* sp. A20, *Sulfolobus tokodaii*, and *Acidianus manzaensis* experienced a genome expansion, *S. acidocaldarius*, *Metallosphaera cuprina*, *M. sedula*, and *Acidianus hospitalis* lost genes in course of their genome diversification. Specifically for the multistrain species, this result corroborated well with the genome size of respective species (average genome sizes are 2.65 ± 0.12, 2.81 ± 0.16, 2.17 ± 0.08, and 2.14 ± 0.13 Mbs for the strains of *S. islandicus*, *S. solfataricus*, *S. acidocaldarius*, and *M. sedula*, respectively). We also identified geographical location-wise gene gains and losses for *S. islandicus* and *S. solfataricus*. In case of *S. islandicus*, population specific to all three major geographical locations experienced net gene gain, whereas subpopulations from Russia experienced less amount of net loss and YNP subpopulation within the United States experienced higher gene gains. Similar kinds of population- and subpopulation-level gene gain–loss dynamics for *S. islandicus* except for Russian subpopulation were reported in an earlier pan-genome–based work (Reno et al., 2009).

It is to be noted here that a similar kind of gene gain–loss pattern can also be seen when we used Sankoff’s algorithm (Supplementary Figure 2). The initial phase of genome shrinkage and subsequent expansion along with dynamic gene gain–loss can also be seen.

The gene sharing pattern within the different members of the family *Sulfolobaceae* as depicted in Figure 1 was found to be in complete corroboration with the gene sharing pattern of the multistrain species, viz., *S. islandicus*, *S. solfataricus*, *S. acidocaldarius*, and *M. sedula*, when the pan-genomes of these species were reconstructed separately (Supplementary Figures 3–6). For example, 2,158 genes are core in nature in *M. sedula*–specific pan-genome (Supplementary Figure 6), and this number is close to the gene number calculated for *M. sedula* taxa–specific node at gene gain–loss tree (node 26 in Figure 1). We can find a smaller number of gene gain–loss after that node for *M. sedula* genomes. In these four pan-genome analyses, the unique genes for different member of any pan-genomic group are also corroborating well with the net gene gain–loss of the respective organism in the terminal node (Figure 1).

Previous studies about the genomic evolution of Archaea, bacteria, and eukaryotes estimated rich ancestral gene content and a prevailing trend of genome streamlining in subsequent lineage adaptation and diversification (Hottes et al., 2013). Some of these models also showed a period of gene gains as they adapt to new environments followed by a longer period of genome streamlining as they diversified (Richards et al., 2014). Our data indicated that in case of *Sulfolobaceae* genome diversification, the trend of genome streamlining was followed by periods of differential gene gains, resulting in an overall genome expansion in some species, whereas there was reduction in others.

Based on a probabilistic model of gene gain and loss, Csuros and Miklos (2009) used the posterior probabilities for ancestral state reconstruction in Archaea, and described gene loss as a major force shaping the archaeal genomes throughout their evolutionary history. Despite this inherent trend, they found that few species experienced overall gene gain overcoming the continuous loss, and *S. solfataricus* was among them, although to a lesser extent than other group of Archaea. Also, they estimated that both the ancestral state node of two genomes of the genus *Sulfolobus* and the terminal node of *S. acidocaldarius* experienced a higher extent of gene loss than gain, whereas the reverse was true for the terminal node of *S. solfataricus*. Thus, our results were similar in part to that study, depicting the general trend of asymmetric gene gains–losses in different species of the family *Sulfolobaceae*. The trend of either overall gene gain or loss was not specific to any particular genus but rather to a particular species or two very closely related species. Any population may successfully thrive into a new environment by inventing or gaining major physiological or metabolic ways. The genomes further diversify by the process of genome streamlining, whereas the advantageous gains or inventions may be retained by population to thrive better.

The process of genome streamlining happens as the genomes diversify by genetic drift given the isolated and disconnected nature of the population characterized by the extreme habitat they thrive in (Whitaker, 2003). Population under significant energy stress may also favor loss of dispensable functions and specialization (Valentine, 2007). Another possibility for genome streamlining is the population-size effects due to the physical isolation (Lynch, 2006), despite the slightly deleterious loss of function. Also, the gene gain events are found to be adaptive in most of the cases (Sela et al., 2016).

### Functional Concurrence for Gene Gain–Loss Events and Metabolic Pathways Diversification

**FIGURE 2:**
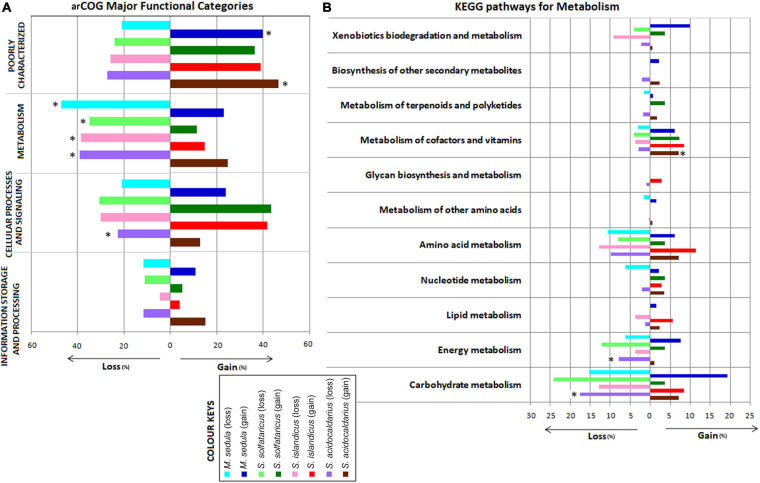
Bar chart showing distribution of major functional categories of Archaeal Clusters of Orthologous Genes (arCOGs) at the species-level gene gain–loss **(A)** and distribution of the KEGG pathways for metabolism at the species-level gene gain–loss **(B)** of the family *Sulfolobaceae*. *Significant difference (*P* < 0.05) in that particular gain–loss event.

We next looked into the impact of gene gain and loss events on cellular functions by examining their representation in arCOGs and KEGG pathway categories from multistrain species, viz., *S. islandicus*, *S. solfataricus*, *S. acidocaldarius*, and *M. sedula*. As these species contain multiple strains, more robust gene gain–loss estimation is possible at the respective species node. Interestingly, the representation from arCOG functional category “Metabolism” was found to be significantly higher (*P* < 0.001) in loss events than the gain ones for all four species. We found significantly higher representation in gene loss than gain for the “Cellular Processes and Signaling” category only for *S. acidocaldarius* (Figure 2A). Further analysis on the KEGG pathway enzymes also followed a similar trend as in the case of arCOG analysis (Supplementary Figure 7). The functional concurrence in the loss events of “Metabolism”-related genes was further looked in more detail. We found that there were mostly replacements of genes for the categories “Carbohydrate metabolism,” “Energy metabolism,” “Amino acid metabolism,” and “Metabolism of cofactors and vitamins” for all these four species (Figure 2B). Moreover, within these pathway categories, *S. acidocaldarius* showed significantly higher gene loss for “Carbohydrate metabolism” and “Energy metabolism,” whereas there was significantly higher gene gain for “Metabolism of cofactors and vitamins.”

The metabolic potential of different *Sulfolobaceae* genomes differs considerably. From their metabolic preferences, it was reported that *Sulfolobus/Saccharolobus* species are mixotrophs growing chemolithoautotrophically or heterotrophically (Keeling et al., 1998). With chemolithoautotrophic metabolic features, these genomes can acquire their necessary carbon from environmental carbon dioxide, as well as they can also use inorganic compounds such as nitrogen and sulfur as their source of energy (Tang et al., 2011). On the other hand, because of their heterotrophic metabolic feature, *Sulfolobus/Saccharolobus* genomes can obtain their energy from organic compounds (Stark et al., 2017). The use of substrates as carbon source varies in *S. solfataricus* and *S. acidocaldarius* strains; the first one can use a wide range of compounds as carbon source including polysaccharides, disaccharides, hexoses, pentoses, aldehydes, alcohols, sugar acids, amino acids, peptides, and tryptone, whereas the latter have a comparatively restricted range of substrate preferences than *S. solfataricus* (Dai et al., 2016; Stark et al., 2017). Another genus *Metallosphaera* contains genomes that are obligate aerobes (Wheaton et al., 2015). The members of this genus are facultative chemolithoautotrophs following autotrophic carbon dioxide fixation pathways with a wide range of inorganic substrate choices. *M. cuprina* is the only reported genome from this genus, which is heterotrophic and shows significant substrate preference for their growth (Wheaton et al., 2015). The genus *Acidianus*, whose members are facultative anaerobes, are also capable of chemolithoautotrophy and can reduce and oxidize elemental sulfur, depending on oxygen availability (Wheaton et al., 2015).

This metabolic dynamicity of different constituent species of family *Sulfolobaceae* may be attributed toward the acquisition or loss of genes mostly from different metabolic pathways in course of their genome divergence. In order to understand in more detail about the repertoire of metabolic pathway genes and their relation with genome divergence, we carried out detailed analysis about the three major metabolic pathways in *Sulfolobaceae*: CCM, sulfur metabolism, and nitrogen metabolism, by considering all the 30 organisms under study.

### Central Carbohydrate Metabolism

Analyses of KEGG enzymes belonging to autotrophic CO_2_ fixation pathways, i.e., reverse ribulose monophosphate pathway (RuMP), citric acid cycle (TCA) along with the glyoxylate shunt, 3-hydroxypropionate/4-hydroxybutyrate (HP/HB) cycle, dicarboxylate/4-hydroxybutyrate cycle (DC/HB), C3/C4 conversions, as well as the heterotrophic pathways such as Embden–Meyerhof–Parnas (EMP) pathway, branched Entner–Doudoroff (ED) pathway (both non-phosphorylative and semiphosphorylative mode), and glycogen and trehalose metabolism for *Sulfolobaceae* genomes, were performed to understand the impact of gene gain–loss on each of these pathways.

Almost all the enzymes in the reverse RuMP pathway were found to be the core in *Sulfolobaceae* family. Only exception was the ribose 5-phosphate isomerase A (RPI, K01807), which helps in the formation of ribose 5-phosphate from ribulose 5-phosphate (Ru5P), which was found to be lost from *S. solfataricus* strains SULA, SULB, SULC, and *S. tokodaii*. In case of the oxidative TCA cycle, all the enzymes were core in nature except for *A. hospitalis*, where the alpha and gamma subunits of pyruvate ferredoxin oxidoreductase (porA, K00169 and porG, K00172) were lost. The functional importance of these subunits would be important for further studies. The enzyme isocitrate lyase (aceA, K01637) was lost from several genomes both at species and genus levels: *S. tokodaii* and *S. solfataricus* strains from Yellowstone National Park and both the genera *Acidianus* and *Metallosphaera*. The enzyme malate synthase (aceB, K01638) that acts on glyoxylate to produce malate and enters into TCA cycle was found to be specifically gained by *M. sedula* genomes. But we have found that *M. sedula* genomes appear to be incapable of producing glyoxylate because of the absence of enzyme aceA in them. It is prominent from the present study that the glyoxylate shunt pathway is not core for *Sulfolobaceae*.

The enzymes required to carry out both HP/HB and DC/HB cycles were core for *Sulfolobaceae*, pointing toward the presence of these autotrophic carbon fixation pathways in this family. In case of *M. sedula* strains, two extra subunits of fumarate hydratase (fumA, K01677 and fumB, K01678) were found to be gained probably during the process of their speciation (Supplementary Table 3 and Supplementary Figure 8).

Again all the enzymes involved in C3/C4 conversions were found to be core in nature, except phosphoenolpyruvate carboxykinase (PCK, K01596), which was specifically lost from *A. hospitalis*. C3/C4 conversions were also found to be a common property for the members of family *Sulfolobaceae* (Supplementary Table 3 and Supplementary Figure 8).

It is well known that the genomes present in the *Sulfolobus* genus can metabolize glucose and galactose by the non-phosphorylative variant of the ED pathway (Dai et al., 2016). It was also reported that in *S. solfataricus* the ED pathway is imperative for glucose and galactose metabolism (Lamble et al., 2005). Here we found that the non-phosphorylative option of the ED pathway was entirely present in the genera *Sulfolobus/Saccharolobus*, except *S. tokodaii*, and was truncated or incomplete for the genera *Acidianus* and *Metallosphaera*. The incompleteness of this pathway was due to the loss of the key enzyme glucose dehydrogenase/aldose 1-dehydrogenase (ssgdh, K18125) by both genera *Acidianus* and *Metallosphaera* and species *S. tokodaii*. In addition, more gene losses were observed for genus *Acidianus* (KDG aldolase, kdpgA, K11395) and specifically for *A. hospitalis* (two subunits of glyceraldehyde dehydrogenase—cutA, K18020, and cutB, K18021). A semiphosphorylative ED pathway also was reported to be present in *S. solfataricus* as a substitute glucose metabolism pathway (Ahmed et al., 2005). As both the steps involving the enzymes ssgdh and kdpgA are common in non-phosphorylative and semiphosphorylative ED pathway, the semiphosphorylative ED pathway was also incomplete for the genera *Acidianus* and *Metallosphaera* and *S. tokodaii*. Moreover, enzymes, which are exclusive for semiphosphorylative ED pathway such as KDG kinase (sskdgK, K18126) and glyceraldehyde-3-phosphate dehydrogenase (gapN, K18978), were lost from the genus *Acidianus*.

We further found complete EMP pathway for gluconeogenesis to be present in family *Sulfolobaceae*, except the interconversion of fructose 6-phosphate and glucose 6-phosphate. The enzyme glucose-6-phosphate isomerase (PGI, K15916) involved in the aforementioned conversion showed gains in species *S. islandicus*, *S. solfataricus*, and *Sulfolobus* sp. A20 and genus *Acidianus* and subsequent loss by few members of species *S. islandicus* and *S. solfataricus*. Glucose 1-phosphate formed in EMP pathway is converted to glycogen by starch synthase (glgA, K00703) that was found to be present for glycogen and trehalose metabolism in all genomes. This glycogen is again reversibly converted to glucose 1-phosphate by the enzyme glycogen phosphorylase (PYG, K00688) that was found to be lost from strain *S. islandicus* Y.N.15.51; species *S. acidocaldarius* and *Sulfolobus* sp. A20 and genera *Acidianus* and *Metallosphaera* signifying the absence of the glycogen to glucose 1-phosphate conversion for these genomes. Glycogen is also converted to glucose by glucoamylase (SGA1, K01178), a core enzyme for *Sulfolobaceae*. Glycogen, produced as a carbon storage compound (Quehenberger et al., 2017), is also a source for trehalose derived from the TreY/TreZ pathway in *Sulfolobus* (Maruta et al., 1996). Our study found that the enzyme (1–>4)-alpha-D-glucan 1-alpha-D-glucosylmutase (TreY, K06044) was gained by the genus *Acidianus*, signifying trehalose formation via the TreY pathway in them. On the other hand, the enzyme maltooligosyltrehalose trehalohydrolase (TreZ, K01236) was lost from *M. sedula*; thus, they did not have any of the TreY and TreZ pathways. Another unique trehalose synthesizing pathway is the unidirectional TreT pathway found in the hyperthermophilic phylum *Crenarchaeota*. We found trehalose synthase (TreT, K13057) to be the core pathway for *Sulfolobaceae* (Supplementary Table 3 and Supplementary Figure 8).

In conclusion, the enzymes involved in the four main autotrophic CO_2_ fixation pathways were almost core in nature for the family *Sulfolobaceae*. Furthermore, the TCA cycle, except the glyoxylate shunt, was also core for them. But the enzymes involved in the heterotrophic cycles were mostly lost from the genera *Acidianus* and *Metallosphaera* and the strain *S. tokodaii*.

Further, we looked into the chemolithotrophic metabolic pathways for *Sulfolobaceae* genomes, where they use inorganic compounds such as sulfur and nitrogen as the source of energy.

### Sulfur Metabolism

In order to get an insight into the relationship of sulfur metabolism capability and evolutionary divergence of *Sulfolobaceae*, hierarchical clustering based on both abundance and presence/absence of sulfur metabolic pathways genes was performed (Supplementary Table 4 and Supplementary Figure 9). Three enzymes, viz., cysteine synthase (cysK, K01738), cystathionine gamma-synthase (metB, K01739), and sulfide quinone oxidoreductase (SQR, K17218), were present in all 30 *Sulfolobaceae* genomes with multiple copies in some cases. SQR can catalyze the oxidation of hydrogen sulfide into polysulfide (Brito et al., 2009), whereas cysK and metB help to produce cysteine from sulfide, which is further used for cysteine and methionine metabolism (Ravanel et al., 1998). The enzymes sulfite reductase (NADPH) hemoprotein beta-component (cysI, K00381), phosphoadenosine phosphosulfate reductase (cysH, K00390), sulfite dehydrogenase (soeB, K21308), sulfate adenylyltransferase (sat, K00958), thiosulfate/3-mercaptopyruvate sulfurtransferase (TST, K01011) were found to be present in most of the *Sulfolobaceae* genomes. cysI, cysH, and sat enzymes were lost by *A. hospitalis* and *M. cuprina*, whereas soeB enzyme was lost by *S. solfataricus* P1 and *A. manzaensis*. cysH was also lost from *Sulfolobus* sp. A20, and TST was lost from the genus *Acidianus*. Multiple copies of cysH were found for *S. acidocaldarius* strains N8, DSM 639, and Ron12/I and *S. islandicus* strains REY15A and L.S.2.15. In addition, multiple copies of TST were present for *M. sedula* strains and most of the *Sulfolobus/Saccharolobus* genomes, except *S. acidocaldarius*. All these aforementioned five enzymes help in the conversion of hydrogen sulfide into sulfite with the successive transformation of the sulfite into sulfate producing ATP by phosphorylation (Dai et al., 2016). Gene encoding sulfate adenylyltransferase subunit 1 (cysN, K00956) was gained solely in *A. hospitalis*. Another important enzyme sulfur oxygenase/reductase (SOR, K16952) for sulfur oxidation in Archaea was lost by all members of *Sulfolobus/Saccharolobus*, except *S. tokodaii* and genus *Metallosphaera* (Dai et al., 2016). Enzyme thiosulfate reductase (phsA, K08352) was gained only by genus *Acidianus*. Multiple copies of doxA (K16936) were present in *S. solfataricus* strains, whereas multiple copies of doxD (K16937) were found in almost all members of *Sulfolobaceae*, except *S. acidocaldarius* SUSAZ and *Sulfolobus* sp. A20. doxDA enzymes are homologs of the TQO (thiosulfate: quinone oxidoreductase), which plays a key role in the transformation of thiosulfate into tetrathionate (Liu et al., 2012). Subunits of the enzyme for sulfur reductase, viz., sulfur reductase molybdopterin subunit (sreA, K17219), sulfur reductase FeS subunit (sreB, K17220), and sulfur reductase membrane anchor (sreC, K17221), are having a significant part in the formation of hydrogen sulfide via reduction of elemental sulfur. These enzymes were primarily gained by *S. islandicus*, *S. solfataricus*, and *A. manzaensis*. Later sreA and sreC were lost from *S. solfataricus* strains isolated from Yellowstone National Park. The only enzyme that helps in the sulfite formation from methane–sulfonate is toluene monooxygenase system ferredoxin subunit (tmoC, K15762) that was found to be lost from the genus *Metallosphaera* and species *A. hospitalis*. Among the *Sulfolobus* genomes, tmoC was lost from all *S. acidocaldarius* strains, *S. tokodaii*, *S. islandicus* M.14.25, *S. islandicus* M.16.27, *S. islandicus* Y.G.57.14, and *S. islandicus* LAL14/1 (Supplementary Table 4 and Supplementary Figure 9).

### Nitrogen Metabolism

In case of nitrogen metabolic pathways enzymes, two subunits of carbamoyl-phosphate synthase (carB, K01955 and carA, K01956) were found in all the 30 *Sulfolobaceae* genomes (Supplementary Table 5 and Supplementary Figure 10). Two other enzymes glutamate dehydrogenase (gdhA, K00261) and glutamine synthetase (glnA, K01915) had multiple copies in some of the genomes. The above results point toward the fact that all the genomes of *Sulfolobaceae* had a common tendency of using ammonia instead of nitrate, urea, cyanate, or formamide, as a source of nitrogen for the glutamate, glutamine, and carbamoyl-phosphate synthesis.

The abundance-based cluster analysis showed that two *S. islandicus* strains each from Iceland (REY15A and LAL14/1) and Russia (M.14.25 and M.16.27) were clubbing together. These strains specifically gained four subunits of nitrate reductase, i.e., narG (alpha subunit, K00370), narH (beta subunit, K00371), narJ (delta subunit, K00373), and narI (gamma subunit, K00374), and thus, they may be capable of nitrate utilization. To be mentioned here, the nitrate/nitrite transporter (nark, K02575) enzyme, primarily present in all *Sulfolobaceae* genomes, was lost from genera *Sulfolobus/Saccharolobus*, species *A. hospitalis*, and *M. cuprina* and then independently gained by the aforementioned four *S. islandicus* strains. The enzyme formamidase (K01455) was only gained by *S. islandicus* HVE10/4, and cyanate lyase (cynS, K01725) was gained by *A. manzaensis* and *S. tokodaii*, suggesting a much wider range of nitrogen sources, such as cyanide and cyanate, for these genomes (Supplementary Table 5 and Supplementary Figure 10).

The detailed analysis of gene gain–loss patterns among the three major metabolic pathways of *Sulfolobaceae* genomes showed that CCM and sulfur metabolism potential almost followed their core/pan-genome diversification pattern. As in case of their genome divergence, within any genus, these two metabolic pathway profiles varied considerably. The repertoires of CCM pathway enzymes were quite distinct for two species of genus *Acidianus*, depicting the impact of gene acquisition and loss in course of their genome divergence. Also, the effect of gene losses was more pronounced in these two pathway genes for all the strains of the species *S. acidocaldarius*, *S. tokodaii*, and *A. hospitalis*. On the other hand, genes involved in nitrogen metabolism were diversely distributed in *Sulfolobaceae* genomes, indicating several independent gene acquisition and loss events irrespective of genome diversification. In case of *S. solfataricus* and *S. acidocaldarius*, this pathway genes seemed to be more conserved as evidenced by their repertoire in isolates from various geographical regions, whereas this was not the scenario for *S. islandicus* genomes and genus *Acidianus*. Thus, our analyses suggested that the ability to use carbohydrate and inorganic sulfur in the members of the family *Sulfolobaceae* has coevolved with their genome diversification but not for nitrogen metabolism.

### Core Genome Diversity and Differential Selective Pressure

Apart from the gene gain–loss events and metabolic pathway differentiation, we looked into the evolutionary divergence of core genes by detecting the evidence of potential recombination events and nucleotide diversity. We identified 30 core genes (6% of the total core genes) with recombination signal, along with putative major parents, minor parents, and recombinants in each of these genes. Core genome phylogenetic tree with non-recombinant core genes (489 genes) was found to be congruent (K tree score = 0.0015) (Soria-Carrasco et al., 2007) with that of previous core genome tree, implying undetectable impact of recombination events on core genome divergence as a whole. As mobile genetic elements influence HGT as well as transposition, it is apparent that less susceptibility to the mobile genetic elements for *S. acidocaldarius* strains in comparison to *S. islandicus* and *S. solfataricus* strains resulted in higher preservation in *S. acidocaldarius* genomes (Brügger et al., 2002; Chen et al., 2005; Redder and Garrett, 2006; Quehenberger et al., 2017; Wagner et al., 2017). However, previous works demonstrated that to be deficient in mobile genetic elements does not result in the low genomic diversity in *S. acidocaldarius* strains. Instead, they proposed that significant disparity in the intrinsic evolutionary rates in the *Sulfolobus/Saccharolobus* genomes produced comparatively low diversity in the *S. acidocaldarius* strains (Anderson et al., 2017).

**FIGURE 3:**
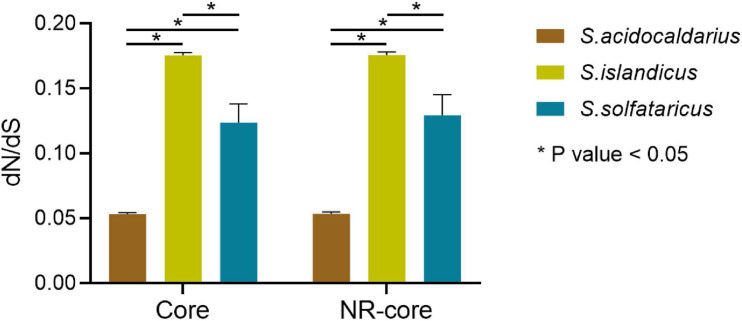
Average dN/dS values for the core genes and non-recombinant core genes belonging to the species with multiple strains (*S. islandicus*, *S. solfataricus*, and *S. acidocaldarius*).

We further analyzed dN and dS values for multistrain species (*S. islandicus*, *S. solfataricus*, *S. acidocaldarius*, and *M. sedula*) for both 519 core genes and 489 non-recombinant core genes. The values of dN, dS, and dN/dS for *S. acidocaldarius* strains were significantly (*P* < 0.05) lower than that for *S. islandicus* and *S. solfataricus* showing their highly conserved nature (Figure 3 and Supplementary Table 6). Similar trend was observed in non-recombinant core genes. We further incorporated five recently available complete genome sequences of *S. acidocaldarius* (NCBI reference Id: CP020360.1, CP020361.1, CP020362.1, CP020363.1, and CP020364.1) and identified the orthologs of 519 core genes from them and again calculated the dN/dS ratio. For these nine strains of *S. acidocaldarius*, the average dN/dS value of 519 core genes was found to be 0.053, which is again significantly (*P* < 0.05) lower than that of *S. islandicus* and *S. solfataricus*. The selection efficiency can plausibly be quantified by the core genome-wide dN/dS ratio, where a lower dN/dS ratio indicates a stronger purifying selection on an average. Thus, it can be inferred that a strong purifying selection was associated with *S. acidocaldarius* strains from different geographical locations. On the other hand, we found no changes at the nucleotide level of the core genes of the six *M. sedula* strains under study, signifying their absolute resemblance at the core-gene level probably due to their laboratory derived origin (Ai et al., 2015, 2016).

A previous study also reported lower nucleotide divergence in *S. acidocaldarius* genes than in *S. islandicus* (0.15% of *S. islandicus* value) (Mao and Grogan, 2017). Also, the study conducted by Anderson et al. (2017) identified higher conserved core genes from 47 *S. acidocaldarius* genomes (Anderson et al., 2017). This disparity in the extent of the conservation of core genes for *S. acidocaldarius* was quite intriguing. One possible reason could be that the removal of any excess variation might have happened through very recent genetic bottleneck or selective sweep. However, an alternative possibility can be that any excess diversity in response to strongly positive selection pressures was not necessary for the maintenance of lifestyle, survival, and adaptation of *S. acidocaldarius* as a species. In essence, such a high level of conservation was needed owing to strong purifying selection as suggested in this study. Another hypothesis suggested that a more recent purifying event has taken place in the *S. acidocaldarius* population, or their emergence is a recent phenomenon than the *S. islandicus* populations (Mao and Grogan, 2017). Interestingly, in support of our findings, a recent study reported that smaller archaeal genomes experienced stronger purifying selection, as opposed to the common observations of positive selection dynamics in bacterial genomes (Lyu et al., 2017). In another study, epipelagic *Marinimicrobia* has been found to show properties consistent with the hypothesis regarding genome streamlining; i.e., small, compact genomes are correlated with stronger purifying selection pressure (Aylward and Martinez-Gutierrez, 2019). Importantly, within the genus *Sulfolobus*, *S. acidocaldarius* represented the smallest average genome size. This scenario can be explained by the existing theory suggesting strong purifying selection to be operative on prokaryotic genomes characterized by large effective population size so as to retain their compact genomes (Lynch, 2006).

### Lineage and Functional Category–Specific Positive Selection

The branch-site codon model for testing positive selection determined 22 gene sets showing significant (*P* < 0.05; LRT test) adaptive evolution for different strains (Supplementary Tables 7, 8A,B). These 22 genes included 15 genes for *S. islandicus* strains, five genes for both the *S. islandicus* and *S. solfataricus* strains, one gene for both *S. solfataricus* and *S. acidocaldarius* strains, and one gene for *S. acidocaldarius* strains only (Supplementary Tables 8A,B). We then looked into the recombination events for these genes and found that three of them were affected by homologous recombination (Supplementary Table 8A). Interestingly, for all these gene sets, the positive selection signal was present specifically for *S. islandicus* strains. We found the *S. islandicus* strain (marked by “a” in Supplementary Table 8A) to appear as the major parent for one of the recombination events. This result implied that the changes within the positively selected gene pairs might be distributed via the process of homologous recombination.

All these genes were found to have defined functions and further GO enrichment analysis using Comparative GO web server^3^ with *S. islandicus* strain L.S.2.15 as reference yielded significant (*P* < 0.05) enrichment of three molecular functional categories, viz., metal ion binding (GO id: 46872), ATP binding (GO id: 5524), and zinc ion binding (GO id: 8270). Of 22 genes, nine genes were from these three categories. Thus, the positively selected genes were not from random functional categories but were targeted toward particular functions. Among the functional categories of positively selected genes, superoxide dismutase is an up-regulated gene under oxygen-rich conditions in *S. solfataricus* (Sheng et al., 2014), helping the cells resist fatal oxidative stress (Culotta, 2001). Our study revealed that the positively selected changes in the gene pairs representing superoxide dismutase were distributed via the process of homologous recombination.

A comparative genomics study by Zaparty et al. (2010) reconstructed the CCM network where enzymes ribokinase, succinate dehydrogenase, RNA-binding protein, ATP-binding protein, AIR synthase, and different transcription regulating factors were found to be associated with that pathway in *S. solfataricus* (Zaparty et al., 2010). Notably, the present study identified some of these genes to be under positive selection pressures. The gene encoding ribokinase signifies the conversion of ribose 5-phosphate to ribose, a very important enzyme for autotrophic CO_2_ fixation pathway, under positive selection for *S. islandicus* strains. To be noted here, another enzyme succinate semialdehyde reductase (K14465), involved in both HP/HB and DC/HB cycles, was also found to be under positive selection pressure for both *S. islandicus* and *S. solfataricus* strains. Again, the enzyme biotin carboxylase (K01964) was under positive selection pressure and found to be involved in both HP/HB cycle and C3/C4 conversions in these two species. All these three genes were from autotrophic CO_2_ fixation pathways, thus signifying the functional importance of these positively selected genes in autotrophic pathways for the species *S. islandicus* and *S. solfataricus*. In addition, cystathionine gamma-synthase, which helps in the catalysis of the cleavage of cystathionine to generate cysteine, has been found to be under positive selection pressure for *S. islandicus* strains. In Archaea, the major source of sulfur is cysteine for the synthesis of sulfur-containing compounds (Borup and Ferry, 2000), and thus, the enzyme cystathionine gamma-synthase present within the sulfur metabolic pathway may have huge functional importance for the species *S. islandicus*.

In the study of microbial evolution, often the primary emphasis is given to the genome-specific genes acquired by HGT events, whereas the adaptive variations in the core genes get overlooked. Our analysis identified that in the course of genome diversification of the family *Sulfolobaceae*, a considerable amount of core genes representing specific protein functional categories evolved under positive selection pressures in certain species in an asymmetric way. Primarily, the core genes are expected to be conserved and to evolve under strong purifying selection pressures because of the housekeeping roles the respective encoded proteins play in metabolic pathways and physiological activities. Thus, this result is quite interesting, and in the recent past, it was found that the positive selection pressure on core genes was an important mechanism for adaptive evolution of several pathogenic bacteria (Chattopadhyay et al., 2012b). In our analysis, the enrichment of three important molecular functional categories (metal ion binding, ATP binding, and zinc ion binding), as well as the autotrophic CO_2_ fixation pathway enzymes in positively selected genes, further signified the adaptive role of this selection pressure in Archaea. It is to be mentioned here that we could not detect any genes in genera *Acidianus* and *Metallosphaera* to be under positive selection pressure.

This result indicates that for *S. islandicus*, the maximum number of core genes was evolving under positive selection. This positive selection might have played an important role in the strain-level allopatric speciation and adaptation in *S. islandicus* in different geographical locations. Also, several core genes were under the influence of positive selection for both *S. islandicus* and *S. solfataricus* or their common ancestor, which again experienced a considerable amount of gene gains. Thus, both of these species might be under strong selection pressure in terms of acquiring new genes, as well as new sequence feature in their core genome components.

## Conclusion

Comparative pan-genomics along with concomitant evolutionary analyses of 30 genomes of the family *Sulfolobaceae* revealed asymmetric genome evolution. Our analyses on various evolutionary mechanisms responsible for adaptive genome diversification pointed toward the asymmetric nature of these mechanisms shaping the member genomes of this family. This study enabled us to understand the sources of genome-level variations that exist in both species and genus levels within the family *Sulfolobaceae*. Pan-genomics coupled with gene gain–loss analysis explored the inherent pattern of genome streamlining, followed by waves of differential gene gains, which resulted as genome expansion in some species while reduction in others in comparison to the ancestral state. The considerable amount of genome size variation of the members of any genus may be attributable to these waves of gene acquisitions. At the species level, substantial gene gain was evident for *S. islandicus* and *S. solfataricus*, whereas there was loss for *S. acidocaldarius.* Analysis of these gene gain–loss patterns among the three major metabolic pathways of *Sulfolobaceae* genomes revealed that the CCM and sulfur metabolism potential of its members coevolved with the genome diversification pattern of this archaeal family. The autotrophic CO_2_ fixation pathways, except for the glyoxylate shunt, were nearly conserved in the family *Sulfolobaceae*. But the heterotrophic cycles were mostly lost from both the genera *Acidianus* and *Metallosphaera* and the strain *S. tokodaii* 7. Therefore, the CCM pathway enzymes were relatively different for *Acidianus*, *Metallosphaera*, and *S. tokodaii*, whereas the enzymes involved in sulfur metabolism were essentially lost from the genus *Acidianus*, thereby suggesting the impact of gene gain–loss pattern in course of genome divergence of *Sulfolobaceae*.

The strong purifying selection operating on the relatively reduced genomes of *S. acidocaldarius* conformed to a much higher level of sequence conservation in this species than in others. A considerable amount of core genes of *Sulfolobaceae* was found to evolve under positive selection pressures in a lineage- and functional category-specific manner, as evidenced predominantly in the adaptive evolution of *S. islandicus*. While thriving in the similar niche, the lineages can experience contrasting evolutionary routes leading to differential genome adaptation trail as indicated in our study. The enrichment of important functional categories, viz., metal ion binding, ATP binding, and zinc ion binding in positively selected genes, as well as important CO_2_ fixation pathway genes, further signified the adaptive role of these genes. The genera *Sulfolobus/Saccharolobus* has potential in biotechnological application due to their wide substrate specificity, less contamination possibility, thriving in extreme conditions, and no need for cooling arrangements (Abdel-Banat et al., 2010). Also, the extreme growth conditions of this genus characterized by high temperature and low pH are potentially beneficial for several industries by increasing the substrate solubility (Quehenberger et al., 2017). In this regard, it is important to further assess these positively selected genes for their functional significance by laboratory experiments.

## Data Availability Statement

The original contributions presented in the study are included in the article/Supplementary Material, further inquiries can be directed to the corresponding author/s.

## Author Contributions

RB conceptualized the project, performed the analysis, generated and organized the results. NMC, AL, AG, and DB performed required programming during analysis and data generation. CD and SC added thoughtful suggestions during the work and made critical reading of the manuscript. DHH performed the analysis of gene gain–loss events. SP conceived and coordinated the project and performed data analysis and interpretation. SP and RB wrote the manuscript with inputs from other authors. All the authors read and approved the final manuscript.

## Conflict of Interest

The authors declare that the research was conducted in the absence of any commercial or financial relationships that could be construed as a potential conflict of interest.

## Footnotes

1
http://www.ncbi.nlm.nih.gov/genbank/

2
http://www.sulfosys.com/ccm-pathway-map.html

3
http://www.comparativego.com/
