# Corrigendum: Interplay of various evolutionary modes in genome diversification and adaptive evolution of the family *Sulfolobaceae*

**DOI:** 10.3389/fmicb.2024.1538738

**Published:** 2024-12-16

**Authors:** Rachana Banerjee, Narendrakumar M. Chaudhari, Abhishake Lahiri, Anupam Gautam, Debaleena Bhowmik, Chitra Dutta, Sujay Chattopadhyay, Daniel H. Huson, Sandip Paul

**Affiliations:** ^1^Structural Biology and Bioinformatics Division, CSIR-Indian Institute of Chemical Biology, Kolkata, India; ^2^Academy of Scientific and Innovative Research (AcSIR), Ghaziabad- 201002, India; ^3^Department of Pharmacoinformatics, National Institute of Pharmaceutical Education and Research, Kolkata, India; ^4^JIS Institute of Advanced Studies and Research, JIS University, Kolkata, India; ^5^Institute for Bioinformatics and Medical Informatics, University of Tübingen, Tübingen, Germany; ^6^Cluster of Excellence: Controlling Microbes to Fight Infection, Tübingen, Germany

**Keywords:** thermoacidophilic archaea, pan-genome, genome evolution, positive selection, metabolic pathways

In the published article, there was an error in **affiliation 2**. Instead of “Academy of Scientific and Innovative Research (AcSIR), Ghaziabad, India”, it should be “Academy of Scientific and Innovative Research (AcSIR), Ghaziabad- 201002, India.”

The authors apologize for this error and state that this does not change the scientific conclusions of the article in any way. The original article has been updated.

