# Supplementary material for: Interplay of Various Evolutionary Modes in Genome Diversification and Adaptive Evolution of the Family Sulfolobaceae
Source: Front Microbiol. 2021 Jun 25;12:639995. doi: 10.3389/fmicb.2021.639995 (PMC8267890; doi:10.3389/fmicb.2021.639995)
Supplement: Supplementary file 9 [file Data_Sheet_1.PDF]

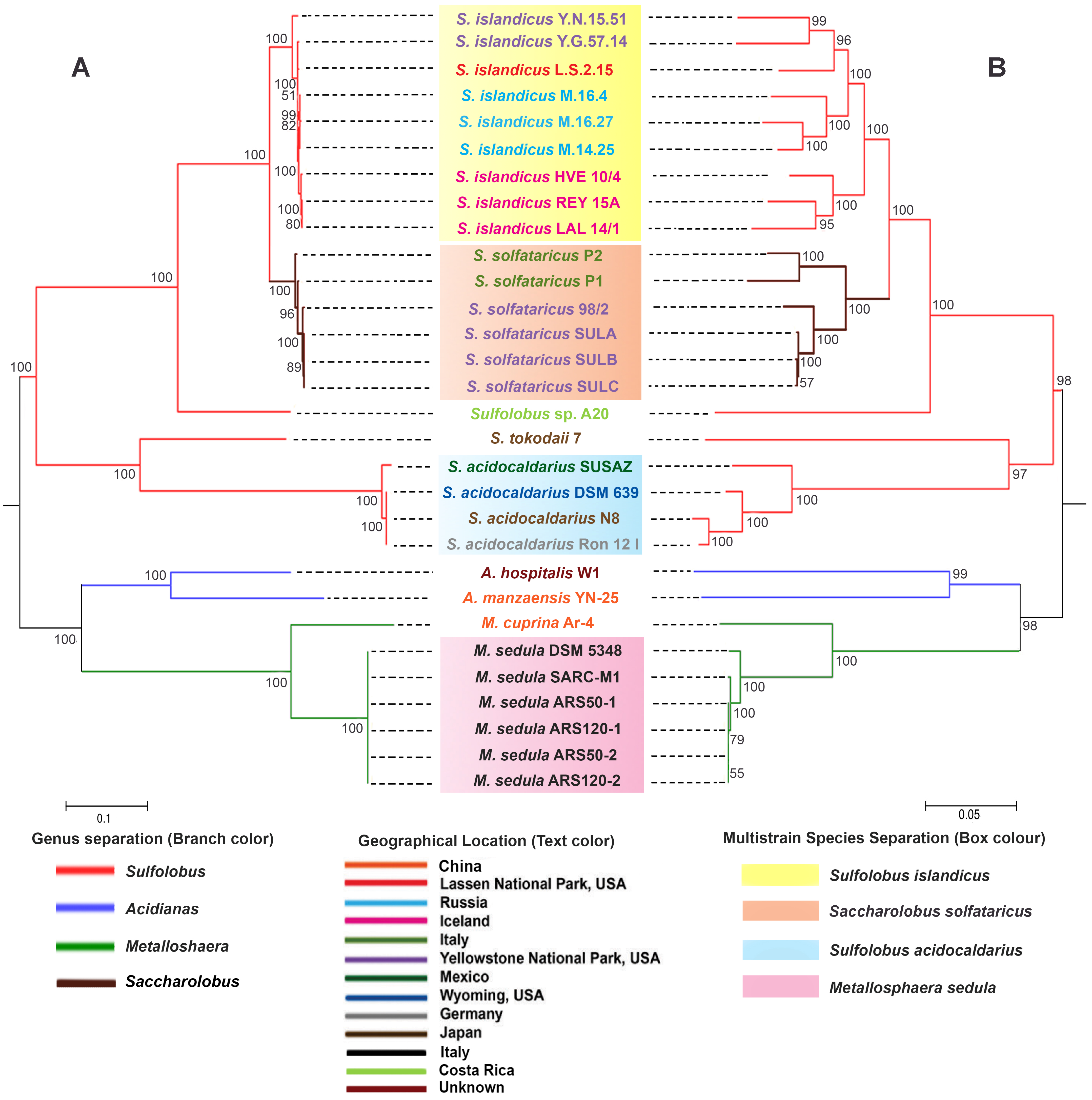

Supplementary Figure 1: The Core Phylogeny (A) and the Pan Phylogeny (B) constructed for the *Sulfolobaceae* family. The Core Phylogeny is based on all genes (including recombinant ones).
