# Supplementary material for: Interplay of Various Evolutionary Modes in Genome Diversification and Adaptive Evolution of the Family Sulfolobaceae
Source: Front Microbiol. 2021 Jun 25;12:639995. doi: 10.3389/fmicb.2021.639995 (PMC8267890; doi:10.3389/fmicb.2021.639995)
Supplement: Supplementary file 10 [file Data_Sheet_2.PDF]

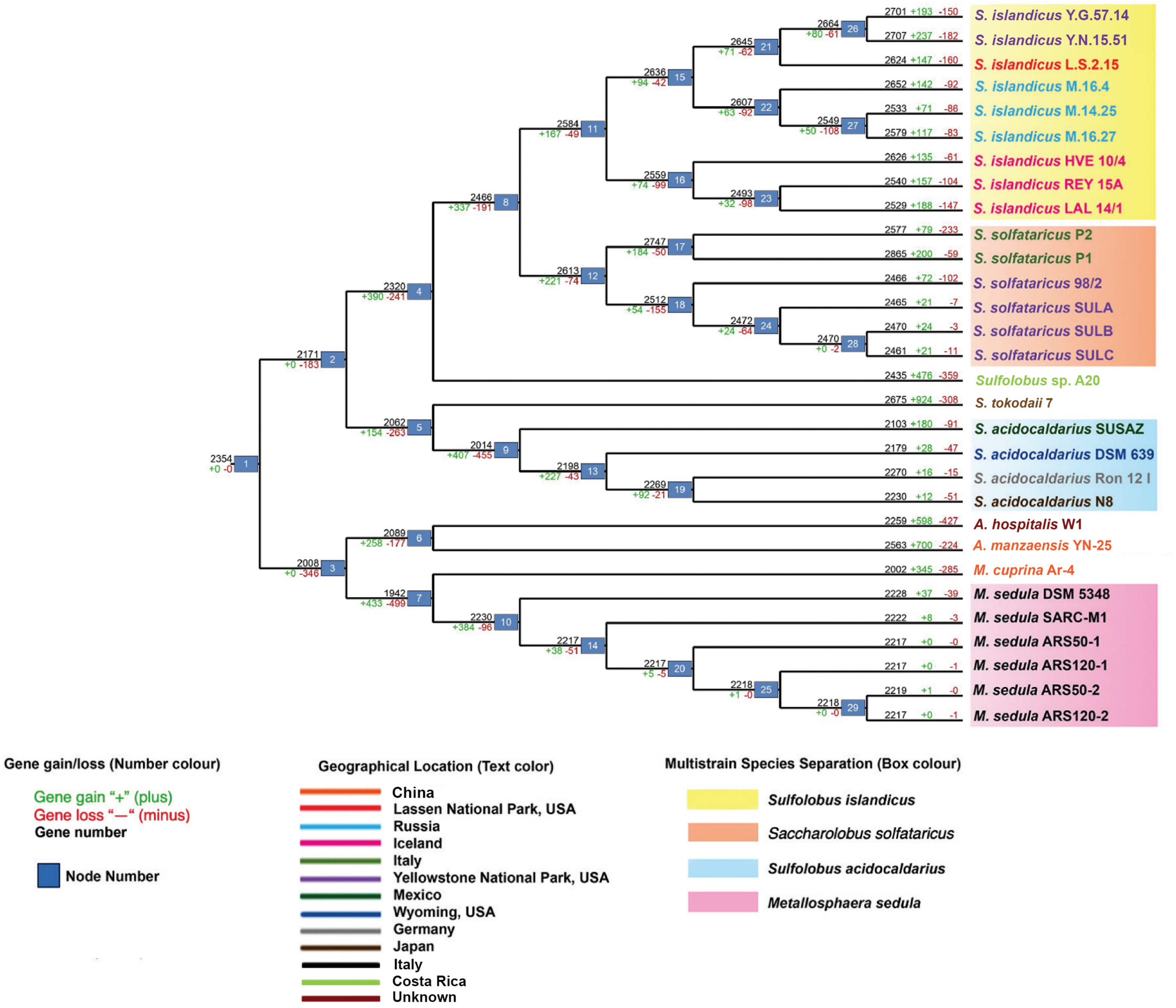

Supplementary Figure 2: Summary of gene gain and gene loss events using Sankoff's algorithm in the family *Sulfolobaceae*, across the phylogenetic tree.
