# Supplementary material for: Interplay of Various Evolutionary Modes in Genome Diversification and Adaptive Evolution of the Family Sulfolobaceae
Source: Front Microbiol. 2021 Jun 25;12:639995. doi: 10.3389/fmicb.2021.639995 (PMC8267890; doi:10.3389/fmicb.2021.639995)
Supplement: Supplementary file 11 [file Data_Sheet_3.PDF]

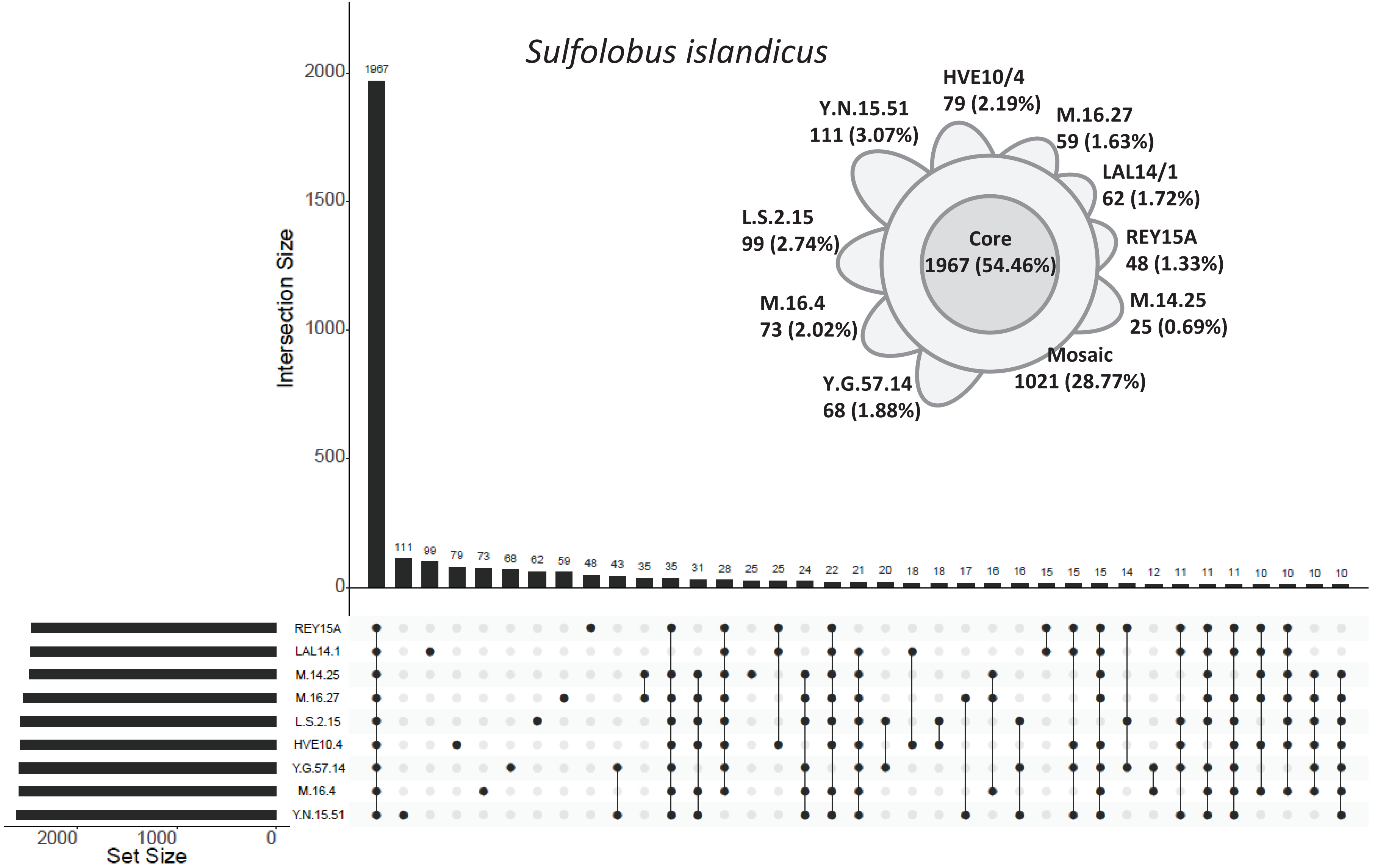

Supplementary Figure 3: UpSet plot of the distribution of protein clusters (50% sequence identity and length coverage) within the *S. islandicus* genomes. For mosaic part only gene sets carrying at least 10 genes are shown. Also the number of gene clusters are shown in core, mosaic and unique groups. UpSet plots were generated using the UpSet Shiny App (https://gehlenborglab.shinyapps.io/upsetr/).
