# Supplementary material for: Interplay of Various Evolutionary Modes in Genome Diversification and Adaptive Evolution of the Family Sulfolobaceae
Source: Front Microbiol. 2021 Jun 25;12:639995. doi: 10.3389/fmicb.2021.639995 (PMC8267890; doi:10.3389/fmicb.2021.639995)
Supplement: Supplementary file 12 [file Data_Sheet_4.PDF]

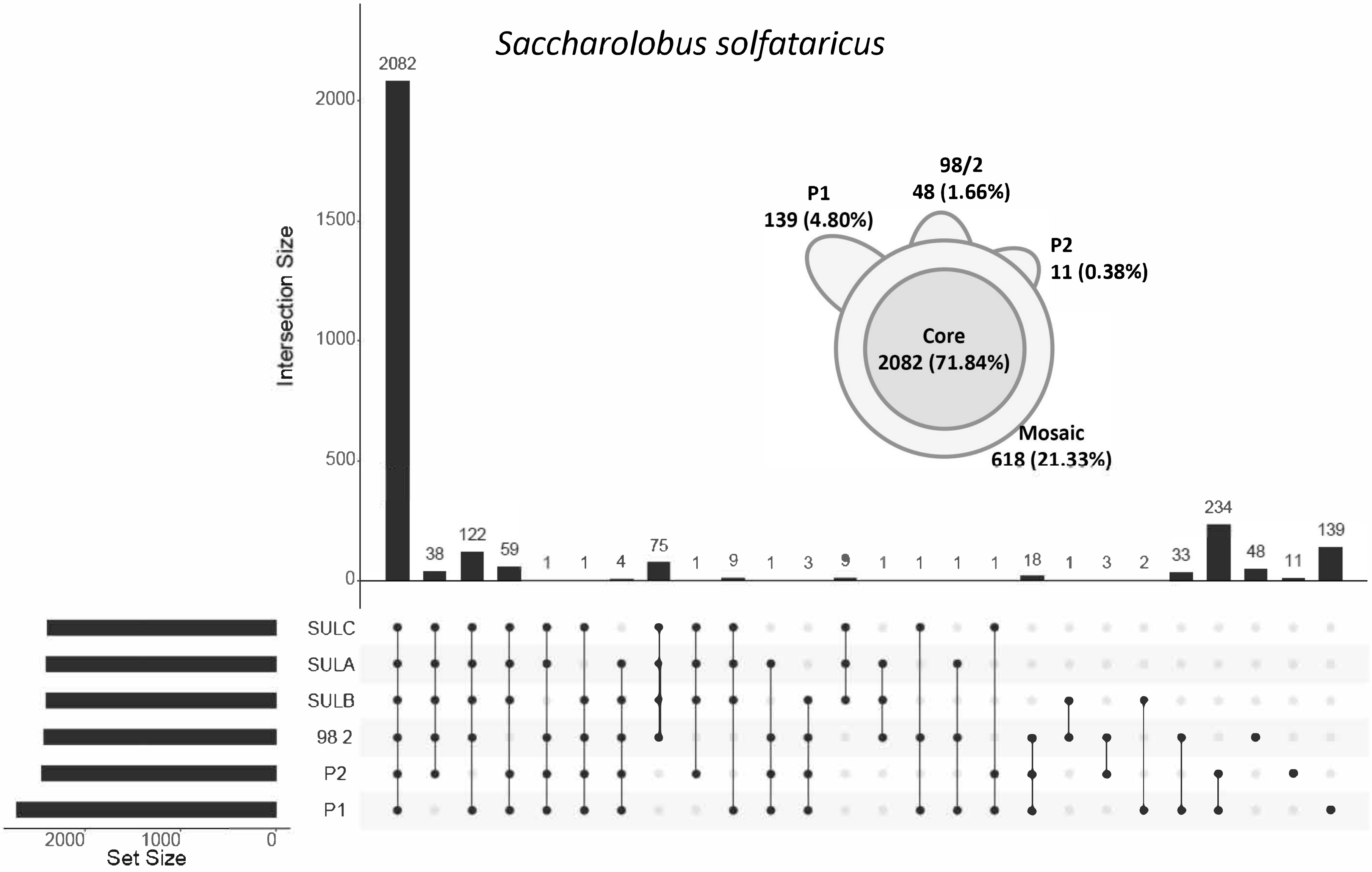

Supplementary Figure 4: UpSet plot of the distribution of protein clusters (50% sequence identity and length coverage) within the *S. solfataricus* genomes. Also the number of gene clusters are shown in core, mosaic and unique groups. UpSet plots were generated using the UpSet Shiny App (https://gehlenborglab.shinyapps.io/upsetr/).
