# Supplementary figures and images for: Interplay of Various Evolutionary Modes in Genome Diversification and Adaptive Evolution of the Family Sulfolobaceae
Source: Front Microbiol. 2021 Jun 25;12:639995. doi: 10.3389/fmicb.2021.639995 (PMC8267890; doi:10.3389/fmicb.2021.639995)

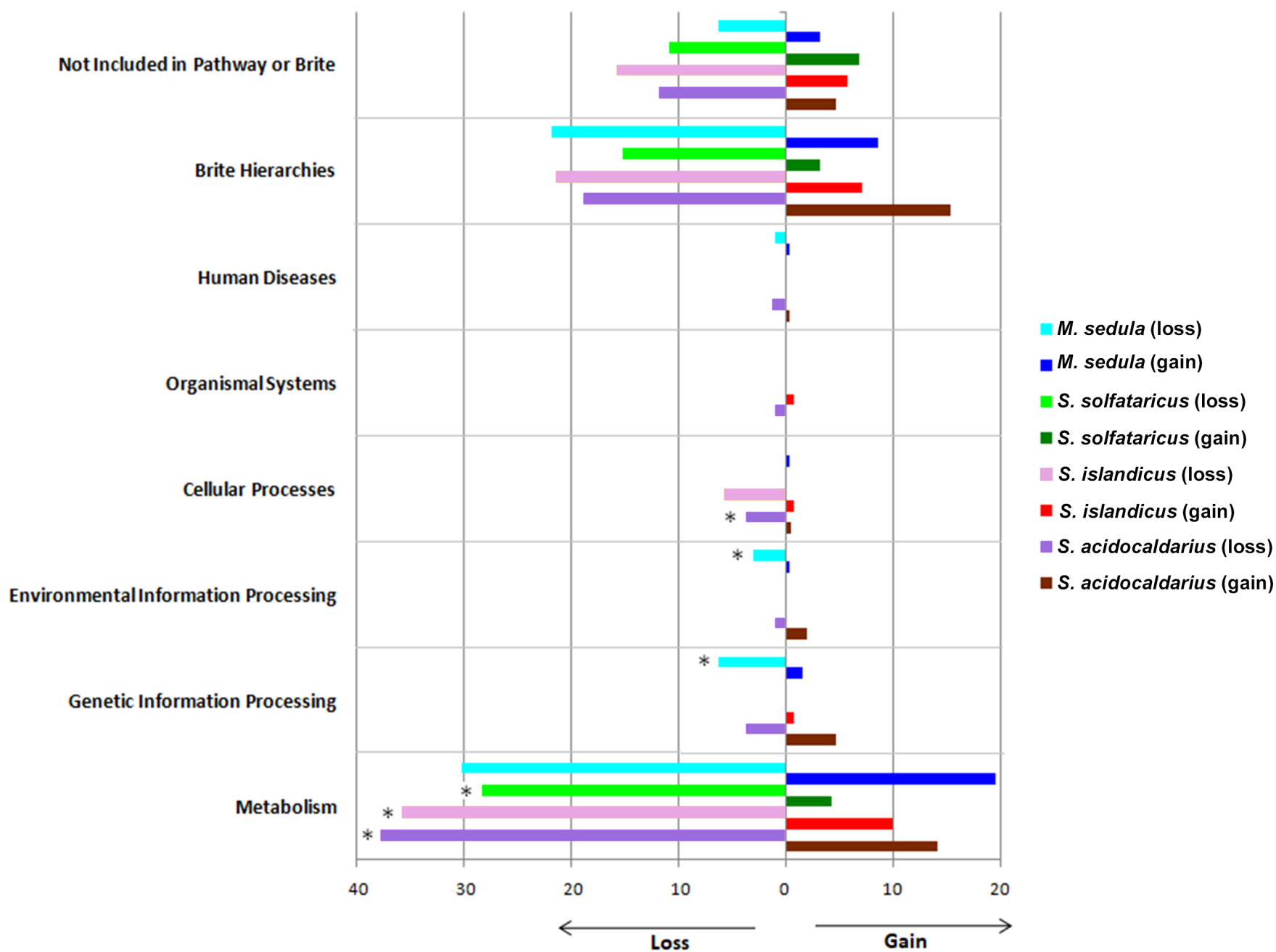

Supplement: Supplementary file 15 [file Data_Sheet_7.PDF]
