# Supplementary material for: Interplay of Various Evolutionary Modes in Genome Diversification and Adaptive Evolution of the Family Sulfolobaceae
Source: Front Microbiol. 2021 Jun 25;12:639995. doi: 10.3389/fmicb.2021.639995 (PMC8267890; doi:10.3389/fmicb.2021.639995)
Supplement: Supplementary file 16 [file Data_Sheet_8.PDF]

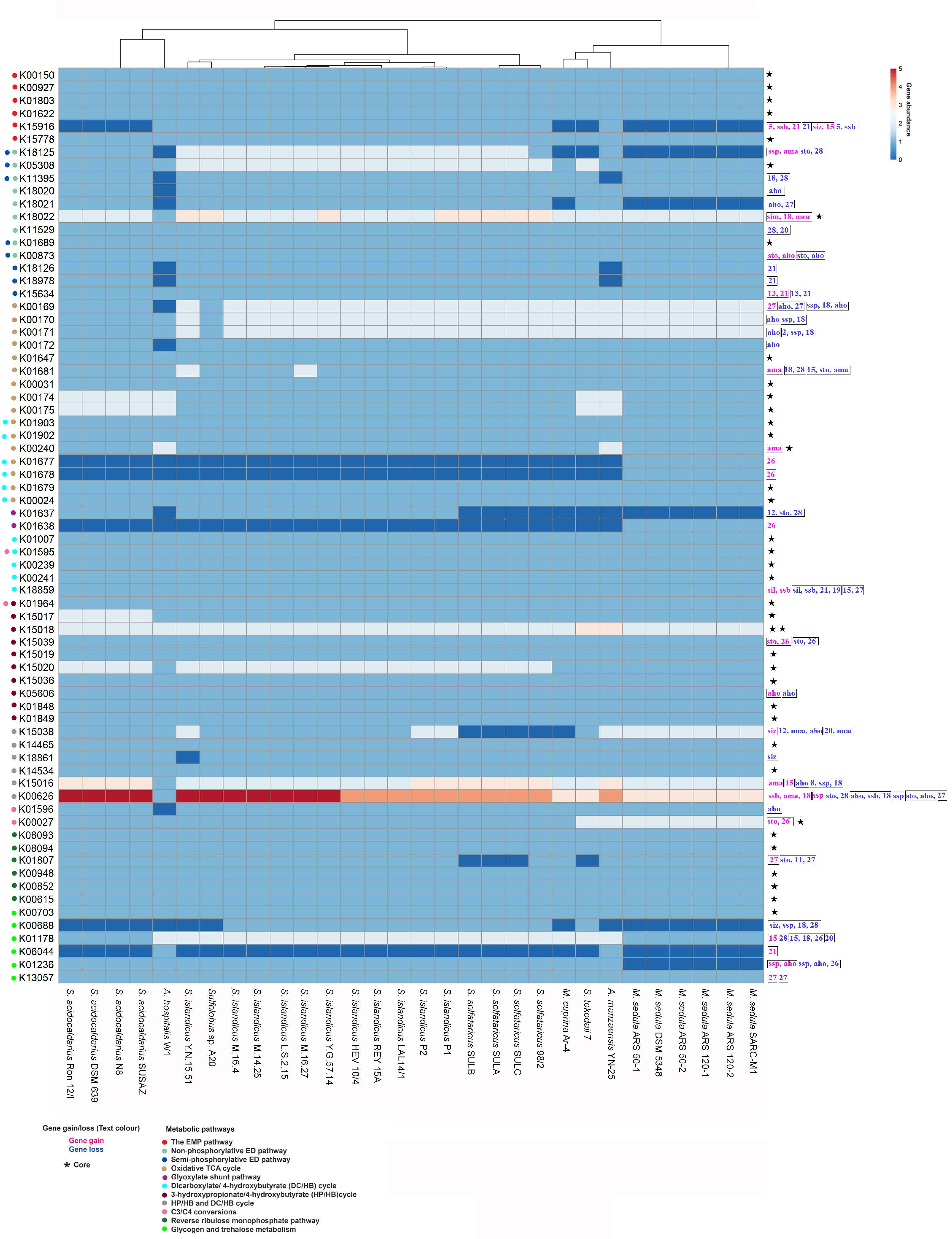

Supplementary Figure 8: Heatmap showing the abundances of 11 'Central Carbohydrate Metabolism' pathway genes in 30 *Sulfolobaceae* genomes. The clustering of the 30 *Sulfolobaceae* genomes shown at the top of the heatmap is based on the abundance values of the CCM pathway genes present within these genomes. The gene clusters are clubbed according to their KEGG ids (left of the heatmap) and presence of any core gene cluster is marked by * (right of the heatmap). The gain-loss events (rectangular boxes) for a gene cluster are also mentioned (right of the heatmap) for any node (Figure 1)/genome (for abbreviation see Supplementary Table 1). For details about gene gain-loss see Supplementary Table 3.
